# Association of skeletal muscle index and intermuscular adipose tissue index measured by non-contrast chest CT with major adverse cardiovascular events in patients with coronary artery disease: a nested case-control study in a Chinese population

**DOI:** 10.3389/fcvm.2026.1917241

**Published:** 2026-08-12

**Authors:** Xu Wu, Haicheng Qi, Ying Chen, Yilong Cai, Xinwei Zhang, Zicheng Zhao, Hui Zhang, Yan Xing

**Affiliations:** 1 Imaging Center, The First Affiliated Hospital of Xinjiang Medical University, Urumqi, Xinjiang, China; 2 Clinical Science Research Department, CT & MR Business Division, Canon Medical Systems, Beijing, China; 3 Department of Oncology, Xinjiang Uygur Autonomous Region Traditional Chinese Medicine Hospital, Urumqi, Xinjiang, China

**Keywords:** coronary artery disease, intermuscular adipose tissue index, major adverse cardiovascular events, plain chest CT, skeletal muscle index

## Abstract

**Objective:**

To investigate the association between chest CT-derived body composition indices and major adverse cardiovascular events (MACE) in coronary artery disease (CAD) patients, and to evaluate their utility for risk stratification.

**Materials and methods:**

This nested case-control study was drawn from a consecutive cohort of 2,226 patients with CAD diagnosed by coronary CT angiography (CCTA) between January 2019 and March 2021. During follow-up, 275 patients developed MACE and were included as cases. From the remaining non-MACE cohort members, 275 controls were selected by simple random sampling, yielding a total of 550 patients. Areas of subcutaneous adipose tissue (SAT), skeletal muscle (SM), intermuscular adipose tissue (IMAT) and visceral adipose tissue (VAT) at the T8 level were segmented on non-contrast chest CT with 3D Slicer, and the corresponding indices and the muscle-to-fat ratio (MSFR) were derived. Associations with MACE were assessed with an inverse-probability-of-sampling weighted Cox proportional-hazards model (robust variance) with pre-specified confounder adjustment, testing of the proportional-hazards assumption, subgroup and interaction analyses, and restricted cubic spline (RCS) analysis.

**Results:**

A total of 550 patients with CAD were enrolled (275 per group). During a median follow-up of 18 months, 275 MACE events occurred. The MACE group had significantly lower skeletal muscle index (SMI) (37.99 vs. 41.27 cm²/m², *P* = 0.003) and MSFR, and higher IMAT index (IMATI) (7.47 vs. 6.21 cm²/m², *P* < 0.001) than the non-MACE group. Higher SMI (HR = 0.636, *P* < 0.001) was independently associated with lower MACE risk, while higher IMATI (HR = 1.994, *P* < 0.001) was independently associated with higher risk. Adding SMI and IMATI to traditional risk factors improved reclassification (IDI = 0.044; NRI = 0.502). Restricted cubic spline analysis showed a non-linear inverse association between SMI and MACE risk, and a non-linear positive association between IMATI and MACE risk.

**Conclusion:**

Higher SMI was independently associated with a lower risk of MACE, whereas higher IMATI was independently associated with a higher risk. Skeletal muscle mass and intramuscular fat infiltration derived from non-contrast chest CT may serve as supplementary indicators for cardiovascular risk stratification in patients with CAD.

## Introduction

Coronary artery disease (CAD) ranks as the most prevalent cardiovascular disorder worldwide. It is associated with high incidence, disability and mortality, placing a substantial public health burden (1, 2). Socioeconomic progress and unhealthy lifestyles have shifted CAD onset to younger age groups (3). Whilst statins and revascularisation therapies have improved patient management, many individuals still carry residual risk of cardiovascular events (4). Major adverse cardiovascular events (MACE) are hard prognostic endpoints and indicate permanent cardiac injury (5). Many MACE events occur in patients stratified as low or moderate risk by conventional risk tools, highlighting limitations within existing risk prediction frameworks (6).

Body composition reflects metabolic function, nutritional status and physiological reserve, with accumulating evidence linking it to cardiovascular outcomes (7, 8). Unlike body mass index (BMI), it separately quantifies the distribution of adipose and skeletal muscle tissue. Excessive fat deposition induces insulin resistance, dyslipidaemia and chronic inflammation, independently accelerating atherosclerosis (9, 10). Likewise, low muscle mass disrupts metabolic homeostasis, raises inflammatory markers and predicts unfavourable cardiovascular prognoses. Raised intermuscular adipose tissue (IMAT) is common among cardiovascular patients and outperforms BMI when discriminating MACE risk (11). Most studies investigating body composition and cardiovascular risk utilise abdominal CT; systematic assessments based on routine plain chest CT remain scarce (8, 12, 13). This research gap holds clear clinical relevance. Plain chest CT is a standard admission investigation for patients with CAD, offering rapid scanning, low radiation doses and no risk of contrast-related adverse reactions. In routine clinical practice, only a small minority of CAD patients undergo abdominal CT. Opportunistic body composition assessment from these routine chest CT scans can cover far larger numbers of CAD patients, with no additional radiation exposure or financial cost (14).

Plain chest CT also enables quantification of epicardial adipose tissue (EAT), a well-validated cardiovascular risk biomarker (15, 16). EAT corresponds to local pericoronary fat depots, whereas the skeletal muscle index (SMI) and IMAT index (IMATI) capture systemic muscle mass and quality. These adipose and muscle compartments influence cardiovascular risk via partially distinct biological pathways. The present study focuses on SMI and IMATI; EAT was not quantified here and will be discussed in greater detail within the Discussion section.

This study utilised body composition indices derived from clinical plain chest CT scans to systematically investigate their association with MACE risk. The aim was to stratify cardiovascular risk using these indices and to provide an evidence-based foundation for early risk screening and personalised clinical intervention in patients with CAD.

## Materials and methods

### Study population

This nested case-control study prospectively enrolled 3,248 patients who underwent plain chest CT at our hospital between January 2019 and March 2021 and were diagnosed with CAD by coronary CT angiography (CCTA). Inclusion criteria were: (1) CAD confirmed by CCTA; (2) age ≥ 18 years; (3) plain chest CT performed; (4) no history of major thoracic surgery or congenital malformations affecting body composition measurement on chest CT; (5) complete baseline clinical data. Exclusion criteria were: (1) conditions affecting body composition (e.g., thyroid disorders, Cushing's syndrome, malnutrition, muscular dystrophy, severe infection, sepsis, active autoimmune disease); (2) hormone therapy, immunosuppressive therapy or parenteral nutrition within the prior 3 months; (3) concurrent malignant tumours, particularly advanced-stage, current radiotherapy/chemotherapy, or distant metastases; (4) severe organ diseases (e.g., chronic heart failure, respiratory failure, hepatic/renal failure, cirrhosis, end-stage chronic kidney disease, severe arrhythmias). A total of 2,226 patients were ultimately included. CAD was defined as ≥ 50% luminal stenosis in at least one major epicardial coronary artery on CCTA, interpreted by experienced cardiovascular radiologists.

Patients were followed up through hospital records, telephone calls and outpatient visits, selected according to individual circumstances. Follow-up began at enrolment and ended at the occurrence of a predefined endpoint, loss to follow-up, or the study end date. Median follow-up was 18.0 months (IQR 13–60 months; range 1–64 months), totalling 1,339 person-years, of whom 275 participants developed MACE. MACE included cardiac death, non-fatal myocardial infarction, non-fatal ischaemic stroke, readmission for heart failure, and unstable angina. Follow-up data recorded the occurrence, timing and aetiology of endpoint events, as well as medication use. Dedicated research staff meticulously recorded and retained complete follow-up records to ensure data traceability.

**Figure 1:**
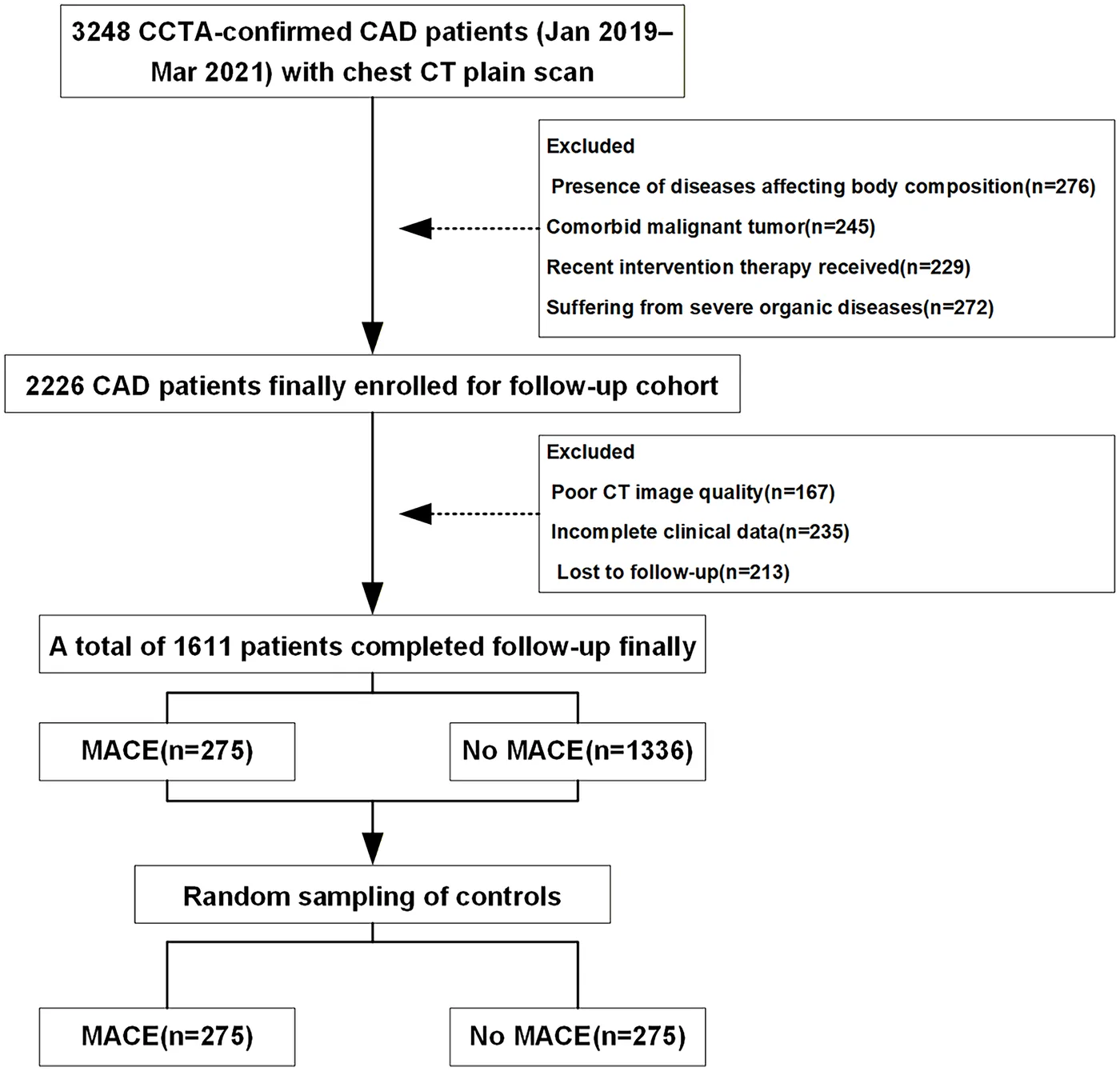
Flowchart of the study population.

Two hundred and thirteen patients (9.6%) were lost to follow-up. After excluding these, those with poor-quality chest CT images, and those for whom image segmentation could not be completed, 275 patients with MACE were identified and included in the case group. To minimise the impact of sample size imbalance on statistical inference, 275 patients who never experienced MACE were selected by simple random sampling from the candidate population to form the control group; no baseline matching was performed. A total of 550 patients were ultimately enrolled. This study was approved by the hospital's Medical Ethics Committee (Ethics Approval No. S-4 220120-02) and adhered to the Declaration of Helsinki; all enrolled patients provided written informed consent. The study flowchart is shown in Figure 1.

### Chest CT scan protocol

In accordance with the European Society of Cardiovascular Radiology (ESCR) guidelines (17), scans were performed using a GE 64-slice CT scanner (LightSpeed VCT; GE; USA). All patients were positioned supine and scanned at the end of deep inspiration, with the scan range extending from the thoracic inlet to the lower margin of the diaphragm, covering the basic thoracic structures (18). Technical parameters were: tube voltage, 120 kVp; tube current, 300 mAs; slice thickness, 5 mm; slice spacing, 5 mm; matrix, 512 × 512.

### Body composition analysis based on chest CT

**Figure 2:**
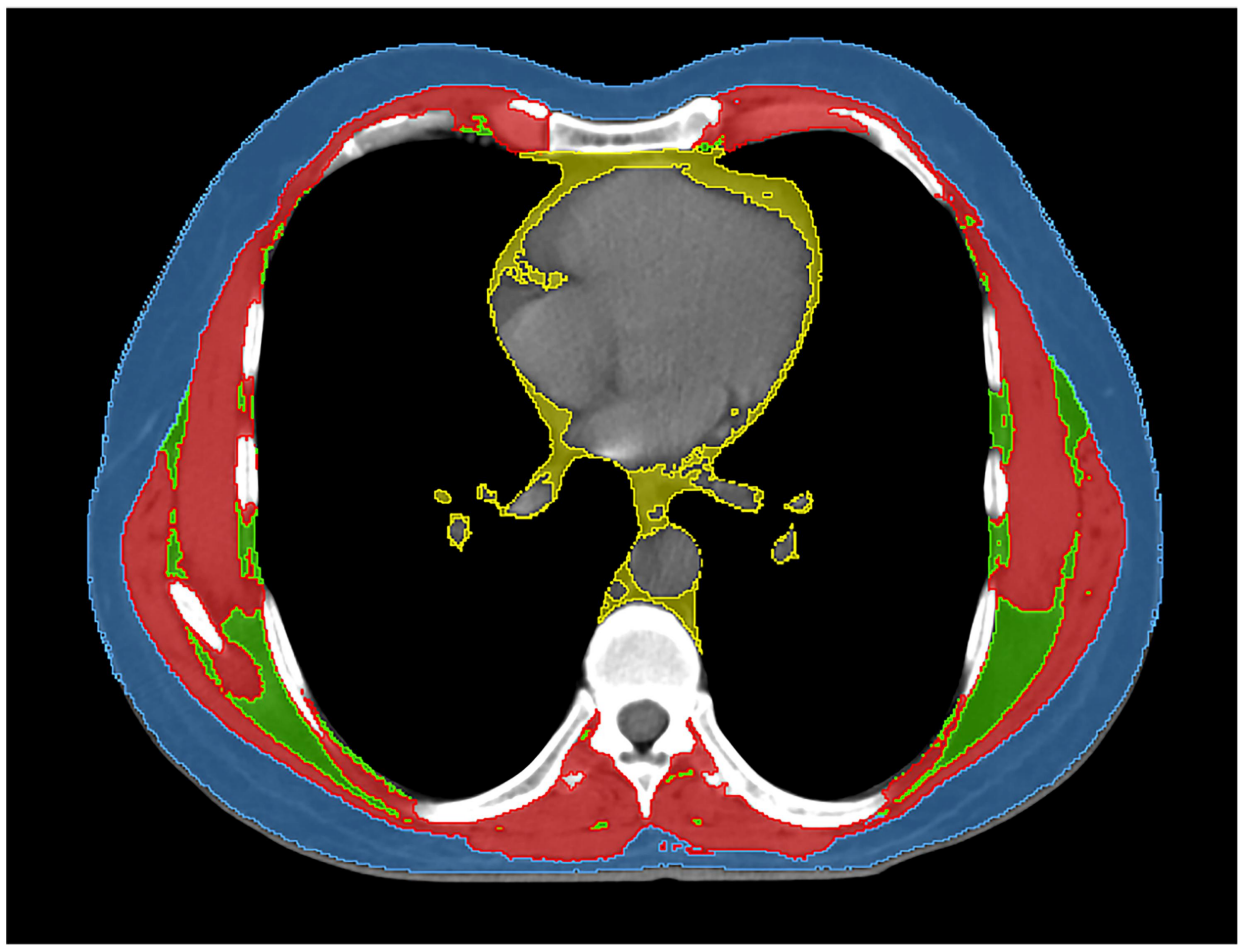
Body composition measurement by computed tomography. Blue indicates SAT, yellow represents VAT, red denotes SM, and green designates IMAT.

The semiautomated analysis software 3D Slicer (version 5.9.0; https://www.slicer.org/) was used to quantitatively analyse subcutaneous adipose tissue (SAT), skeletal muscle (SM), IMAT and visceral adipose tissue (VAT) at the T8 vertebral body level on non-contrast chest CT images (19). The CT value range for SM was set at −29 to +150 HU, whereas that for adipose tissue was set at −190 to −30 HU. SAT was defined as fat between the skin and pectoral muscles; VAT was defined as fat within the thoracic cavity excluding SAT (20); and IMAT referred to adipose tissue between skeletal muscles (21). A representative body-composition segmentation is shown in Figure 2.

Body composition indices were calculated using the tissue areas measured by the software, including the subcutaneous adipose tissue index (SATI), SMI, IMATI and visceral adipose tissue index (VATI). All indices were calculated as tissue area (cm²) divided by height squared (m²). The muscle-to-fat ratio (MSFR) was calculated to reflect the relative proportion of muscle to fat, where MSFR = SMI/SATI. To assess reliability, 50 CT images were randomly selected for consistency testing. Physician 1 and Physician 2 (each with over six years of experience in cardiovascular imaging) independently delineated subsamples to assess inter-observer reliability. Physician 1 re-delineated the subsamples after a three-week interval to assess intra-observer reliability. Reliability was evaluated using the intraclass correlation coefficient. Quantitative analysis of body composition for all baseline CT images was ultimately performed independently by Physician 1.

### Statistical analysis

Analyses were performed with IBM SPSS Statistics 27.0 and R 4.1. Categorical variables are presented as *n* (%) and compared using the chi-square test; continuous variables are presented as mean ± SD (Student's t-test) or median (IQR) (Mann–Whitney U test), as appropriate. Correlations among body composition indices were assessed using Pearson coefficients. Inter- and intra-observer segmentation reliability was assessed using the intraclass correlation coefficient (ICC > 0.75 acceptable). This was a nested case-control study within a defined CAD cohort. Because controls were a random sample of non-MACE cohort members, time-to-event data were analysed with an inverse-probability-of-sampling-weighted Cox model using a robust (sandwich) variance estimator, following established methods for case-cohort sampling (Prentice, 1986; Barlow, 1994). The weight for controls was calculated as the inverse sampling fraction (w = N/*n* = 1,951/275 = 7.09), whereas cases were assigned a weight of 1.

Rather than univariate screening, the multivariable model included a pre-specified set of clinically important confounders (age, sex, smoking, hypertension, diabetes, total cholesterol, HDL-C, LDL-C, BMI, and baseline lipid-lowering and anticoagulant therapy). All secondary analyses, including sex-stratified regression, quartile stratification, subgroup interaction analysis and restricted cubic spline modelling, uniformly adopted this complete pre-defined covariate panel; no *P*-value-based variable screening was performed prior to multivariable adjustment. Multicollinearity was assessed using variance inflation factors (all VIF < 5). The proportional-hazards assumption was tested with scaled Schoenfeld residuals, log-minus-log plots and time-varying coefficient tests; covariates violating the assumption were addressed by stratifying the baseline hazard in a sensitivity model. The incremental value of SMI and IMATI over traditional risk factors was assessed using Harrell's C-statistic, time-dependent AUC (12, 24 and 36 months), integrated discrimination improvement (IDI) and continuous net reclassification improvement (NRI; 1,000 bootstrap resamples). Effect modification was examined by subgroup analyses (age < 65 vs. ≥ 65 years, sex, diabetes, obesity by BMI) with multiplicative interaction terms. Sex-stratified, quartile and subgroup analyses were adjusted for a core set of covariates (age, sex where applicable, hypertension, diabetes and smoking) to preserve statistical power within strata. Restricted cubic splines (RCS) were used to assess non-linear dose-response relationships. All variables were complete, so a complete-case analysis was performed without imputation. Reporting followed the STROBE guidelines. Two-sided *P* < 0.05 denoted statistical significance.

## Results

### Baseline characteristics

**Figure 3:**
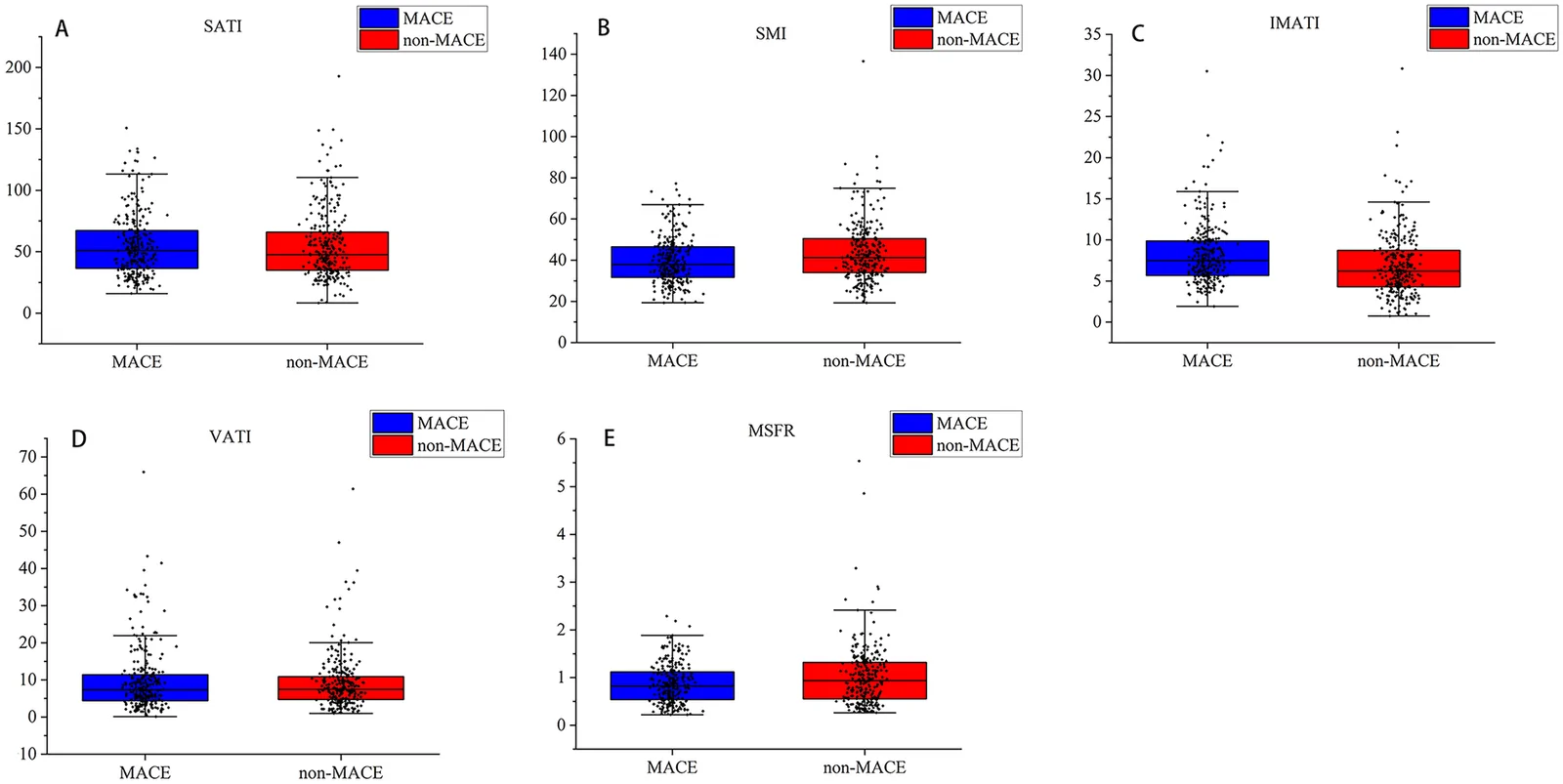
Comparison of body composition indices between the MACE group and non-MACE group. **(A)** SATI; **(B)** SMI; **(C)** IMATI; **(D)** VATI; **(E)** MSFR. Box-and-whisker plots display the distribution of each index in the two groups.

This study included 550 patients with CAD (275 per group). There were no statistically significant differences between the two groups in terms of age or sex (*P* > 0.05). The median follow-up period was 18 months, during which 275 MACE events occurred. MACE events included 23 cases of cardiac death, 46 cases of non-fatal myocardial infarction, 76 cases of non-fatal ischaemic stroke, 33 cases of readmission for heart failure, and 97 cases of unstable angina. The smoking rate was significantly higher in the MACE group than in the non-MACE group (37.8% vs. 26.9%, *P* < 0.05). Conversely, patients without MACE showed significantly greater use of lipid-lowering therapy and anticoagulants (both *P* < 0.001). There were no significant differences between the two groups in SATI (50.96 vs. 47.62 cm²/m², *P* = 0.504) or VATI (7.39 vs. 7.49 cm²/m², *P* = 0.892). In the MACE group, SMI (37.99 vs. 41.27 cm²/m², *P* = 0.003) and MSFR (0.82 vs. 0.94, *P* = 0.025) were significantly lower, whereas IMATI (7.47 vs. 6.21 cm²/m², *P* < 0.001) was significantly higher (Table 1; Figure 3).

**Table 1:** Baseline characteristics of the study population.

Variable	MACE (n = 275)	Non-MACE (n = 275)	*P*-value
Demographics			
Age, years	66.000 (58.0,73.0)	66.00 (57.0,75.0)	0.571
Height,cm	169.00 (160.0,175.0)	169.00 (161.0,174.0)	0.819
Weight,kg	75.00 (66.0,83.0)	73.00 (65.0,83.0)	0.290
BMI, kg/m^2^	26.03 (24.0,29.0)	26.00 (23.8,28.0)	0.170
Male, *n* (%)	186 (67.6)	181 (65.8)	0.651
Smoking, *n* (%)	104 (37.8)	74 (26.9)	**0**.**006**
Drinking, *n* (%)	77 (28.0)	63 (22.9)	0.171
Metabolic components			
Hypertension, *n* (%)	202 (73.5)	187 (68.0)	0.160
Diabetes, *n* (%)	74 (26.9)	62 (22.5)	0.236
Medications			
Lipid-lowering therapy, *n* (%)	22 (8.0)	80 (29.1)	**<0**.**001**
Anticoagulant therapy, *n* (%)	13 (4.7)	37 (13.5)	**<0**.**001**
Laboratory results			
TC, mmol/L	4.05 (3.3,4.8)	4.24 (3.5,4.9)	0.082
TG, mmol/L	1.53 (1.1,2.1)	1.40 (1.0,2.2)	0.381
HDL-C, mmol/L	1.00 (0.8,1.1)	1.01 (0.8,1.2)	0.171
LDL-C, mmol/L	2.60 (1.9,3.3)	2.67 (2.1,3.3)	0.118
SATI,(cm^2^/m^2^)	50.96 (36.5,67.3)	47.62 (34.9,66.0)	0.504
SMI,(cm^2^/m^2^)	37.99 (31.7,46.6)	41.27 (33.9,50.6)	**0**.**003**
IMATI,(cm^2^/m^2^)	7.47 (5.7,9.9)	6.21 (4.3,8.7)	**<0**.**001**
VATI,(cm^2^/m^2^)	7.39 (4.4,11.4)	7.49 (4.7,10.9)	0.892
MSFR	0.82 (0.5,1.1)	0.94 (0.6,1.3)	**0**.**025**

Values are expressed as *n* (%), mean ± standard deviation.

BMI, body mass index; TG, triglycerides; TC, total cholesterol; HDL-C, high-density lipoprotein cholesterol; LDL-C, low-density lipoprotein cholesterol; SATI, subcutaneous adipose tissue index; SMI, skeletal muscle index; IMATI, intermuscular adipose tissue index; VATI, visceral adipose tissue index; MSFR, muscle-to-fat ratio.

Bold values indicate statistically significant results (*P* < 0.05).

### Correlation analysis of body composition indices

A pairwise Pearson correlation analysis revealed that SATI was significantly positively correlated with both IMATI (r = 0.401, *P* < 0.01) and VATI (r = 0.200, *P* < 0.01), and significantly negatively correlated with MSFR (r = −0.677, *P* < 0.01). SMI was significantly positively correlated with both IMATI (r = 0.125, *P* < 0.01) and MSFR (r = 0.439, *P* < 0.01). IMATI was significantly positively correlated with VATI (r = 0.342, *P* < 0.01) and significantly negatively correlated with MSFR (r = −0.291, *P* < 0.01). VATI was significantly negatively correlated with MSFR (r = −0.194, *P* < 0.01). No significant correlations were observed among the remaining indices (*P* > 0.05). Further details are provided in Table 2 and Supplementary Figure S1.

**Table 2:** Correlation analysis of body composition variables.

Variable	SATI	SMI	IMATI	VATI	MSFR
SATI	1	0.022	0.401**	0.200**	−0.677**
SMI	0.022	1	0.125**	−0.038	0.439**
IMATI	0.401**	0.125**	1	0.342**	−0.291**
VATI	0.200**	−0.038	0.342**	1	−0.194**
MSFR	−0.677**	0.439**	−0.291**	−0.194**	1

SATI, subcutaneous adipose tissue index; SMI, skeletal muscle index; IMATI, intermuscular adipose tissue index; VATI, Visceral adipose tissue index; MSFR, muscle-to-fat ratio.

** *P* < 0.01.

### Cox proportional hazards regression model analysis

Across the entire cohort, univariable weighted Cox associations for all candidate variables are presented in Table 3. All inverse-probability-of-sampling weighted Cox proportional-hazards models presented in this subsection were fully adjusted for the unified pre-specified confounder panel: age, sex, smoking status, hypertension, diabetes mellitus, total cholesterol, HDL-C, LDL-C, BMI, lipoprotein(a), baseline lipid-lowering therapy and anticoagulant therapy. Irrespective of univariable significance, a pre-specified set of clinically important confounders was entered into the multivariable model. In this model, higher SMI (HR 0.636, 95% CI 0.510–0.792, *P* < 0.001) was independently associated with a lower risk of MACE, whereas higher IMATI (HR 1.994, 95% CI 1.607–2.475, *P* < 0.001) was independently associated with a higher risk. Older age (HR 0.963 per year), higher BMI (HR 0.871), baseline lipid-lowering therapy (HR 0.222) and anticoagulant therapy (HR 0.414) were also independently associated with a lower risk of MACE, whereas higher lipoprotein(a) was independently associated with a higher risk (HR 1.002 per unit, *P* < 0.001) (Table 3).

**Table 3:** Univariable and multivariable Cox regression for MACE risk .

Variable	Univariable HR (95% CI)	*P*-value	Multivariable HR (95% CI)	*P*-value
Height	1.008 (0.988–1.028)	0.424	—	—
Weight	0.996 (0.985–1.007)	0.489	—	—
Sex	1.110 (0.791–1.557)	0.546	1.341 (0.783–2.296)	0.285
Age	0.991 (0.979–1.004)	0.193	0.963 (0.946–0.979)	**<0**.**001**
Smoking	1.386 (0.987–1.948)	0.060	1.454 (0.932–2.269)	0.099
Drinking	1.138 (0.791–1.640)	0.486	—	—
BMI	0.972 (0.935–1.011)	0.152	0.871 (0.819–0.927)	**<0**.**001**
Hypertension	1.359 (0.954–1.936)	0.089	1.395 (0.932–2.089)	0.106
Diabetes	1.533 (1.055–2.229)	0.025	1.261 (0.810–1.964)	0.305
Lipid-lowering therapy	0.287 (0.174–0.473)	<0.001	0.222 (0.123–0.399)	**<0**.**001**
Anticoagulant therapy	0.496 (0.259–0.951)	0.035	0.414 (0.192–0.891)	**0**.**024**
TC	0.856 (0.734–0.999)	0.048	0.850 (0.564–1.281)	0.437
TG	0.993 (0.911–1.083)	0.879	—	—
HDL-C	0.459 (0.260–0.810)	0.007	0.681 (0.353–1.314)	0.252
LDL-C	0.843 (0.697–1.019)	0.078	0.995 (0.614–1.611)	0.983
Lp(a)	1.001 (1.000–1.001)	0.045	1.002 (1.001–1.003)	**<0.001**
SATI (+1SD)	1.019 (0.875–1.187)	0.806	—	—
SMI (+1SD)	0.775 (0.664–0.904)	0.001	0.636 (0.510–0.792)	**<0**.**001**
IMATI (+1SD)	1.253 (1.076–1.459)	0.004	1.994 (1.607–2.475)	**<0**.**001**
VATI (+1SD)	1.090 (0.942–1.262)	0.247	—	—
MSFR (+1SD)	0.787 (0.669–0.925)	0.004	0.731 (0.500–1.067)	0.104

HR, hazard ratio; 95% CI, 95% confidence interval; BMI, body mass index; TG, triglycerides; TC, total cholesterol; HDL-C, high-density lipoprotein cholesterol; LDL-C, low-density lipoprotein cholesterol; SATI, subcutaneous adipose tissue index; SMI, skeletal muscle index; IMATI, intermuscular adipose tissue index; VATI, visceral adipose tissue index; MSFR, muscle-to-fat ratio.

All multivariable models adjusted for the same pre-specified set of clinically important confounders: age, sex, smoking, hypertension, diabetes, TC, HDL-C, LDL-C, BMI, lipoprotein(a), and baseline lipid-lowering and anticoagulant therapy. SMI and IMATI, the primary exposures, were entered jointly in a single model (all variance inflation factors < 5). MSFR (= SMI/SATI) is mathematically collinear with these muscle and adipose indices; when entered together with SMI and IMATI it showed no independent association with MACE (HR 0.731, *P* = 0.104), indicating that it carries no information beyond the two primary indices. SATI and VATI were not associated with MACE on univariable analysis and are presented for univariable comparison only. Hazard ratios for SMI, IMATI, SATI, VATI and MSFR are per 1-SD increment.

Bold values indicate statistically significant results (P < 0.05).

### Sex-stratified cox proportional hazards regression model analysis

In sex-stratified models adjusted for the same pre-specified clinical covariates, IMATI remained significantly associated with MACE in both men (HR 1.911, 95% CI 1.454–2.511, *P* < 0.001) and women (HR 2.198, 95% CI 1.531–3.155, *P* < 0.001). SMI was significantly protective in men (HR 0.601, 95% CI 0.474–0.764, *P* < 0.001) but did not reach significance in women (HR 0.903, 95% CI 0.462–1.767, *P* = 0.766), a subgroup containing only 89 events; the sex-by-SMI interaction was not significant (*P* for interaction = 0.134), so the two estimates are statistically compatible and the null finding in women most likely reflects limited power. After full adjustment, smoking was associated with MACE in women (HR 6.954, 95% CI 1.419–34.092, *P* = 0.017) but not in men (HR 1.434, 95% CI 0.915–2.247, *P* = 0.115). MSFR was not associated with MACE in either sex (Table 4).

**Table 4:** Sex-stratified multivariable Cox analyses for MACE risk.

Variable	Male HR (95% CI)	*P*-value	Female HR (95% CI)	*P*-value
Age, years	0.968 (0.949,0.988)	**0** **.** **002**	0.939 (0.908,0.971)	**<0**.**001**
Smoking	1.434 (0.915,2.247)	0.115	6.954 (1.419,34.092)	**0**.**017**
Hypertension	1.377 (0.868,2.184)	0.174	1.601 (0.657,3.902)	0.301
Diabetes	1.350 (0.766,2.380)	0.299	1.137 (0.568,2.275)	0.717
BMI, kg/m²	0.880 (0.809,0.957)	**0**.**003**	0.843 (0.769,0.924)	**<0**.**001**
TC, mmol/L	0.894 (0.571,1.402)	0.626	0.821 (0.320,2.109)	0.682
HDL-C, mmol/L	0.782 (0.285,2.143)	0.633	0.553 (0.202,1.517)	0.250
LDL-C, mmol/L	0.974 (0.579,1.637)	0.919	0.970 (0.293,3.204)	0.960
Lp(a), mg/L	1.002 (1.001,1.003)	**<0**.**001**	1.002 (1.000,1.003)	**0**.**018**
Lipid-lowering therapy	0.200 (0.096,0.417)	**<0**.**001**	0.291 (0.098,0.867)	**0**.**027**
Anticoagulant therapy	0.330 (0.127,0.859)	**0**.**023**	0.539 (0.123,2.368)	0.413
SMI(+1SD)	0.601 (0.474,0.764)	**<0**.**001**	0.903 (0.462,1.767)	0.766
IMATI(+1SD)	1.911 (1.454,2.511)	**<0**.**001**	2.198 (1.531,3.155)	**<0**.**001**
MSFR(+1SD)	0.753 (0.511,1.110)	0.152	0.378(0.080,1.784)	0.219

HR, hazard ratio; 95% CI, 95% confidence interval; SMI, skeletal muscle index; IMATI, intermuscular adipose tissue index; MSFR, muscle-to-fat ratio; TC, total cholesterol; HDL-C, high-density lipoprotein cholesterol; LDL-C, low-density lipoprotein cholesterol; Lp(a), lipoprotein(a). Inverse-probability-of-sampling weighted Cox regression with robust variance, fitted separately in men and women and adjusted for the same pre-specified clinical covariates as the main model [age, smoking, hypertension, diabetes, BMI, TC, HDL-C, LDL-C, Lp(a), baseline lipid-lowering and anticoagulant therapy]; sex was omitted as it defines the strata. SMI and IMATI were entered jointly; MSFR was estimated in a separate model additionally adjusted for SMI and IMATI, because MSFR (= SMI/SATI) is collinear with them. *P* for interaction with sex: SMI 0.134, IMATI 0.575, MSFR 0.765.

Bold values indicate statistically significant results (P < 0.05).

### Adjusted association analysis of MACE risk by quartile stratification of body composition indices

After stratifying each index by quartile and adjusting for the same pre-specified confounders, the risk of MACE decreased across increasing SMI quartiles (Q4 vs. Q1: HR 0.431, 95% CI 0.227–0.818, *P* = 0.010; *P* for trend = 0.010) and increased across increasing IMATI quartiles (Q4 vs. Q1: HR 5.237, 95% CI 2.908–9.431, *P* < 0.001; *P* for trend < 0.001). No MSFR quartile was significantly associated with MACE once SMI and IMATI were accounted for (Q4 vs. Q1: HR 0.435, 95% CI 0.157–1.209, *P* = 0.110; *P* for trend = 0.089) (Table 5). These findings were consistent with the multivariable Cox proportional hazards regression analysis of the continuous indices.

**Table 5:** Sensitivity analysis of quartiles association of SMI, IMATI and MSFR with MACE using adjusted Cox regression .

Variable	Group	HR (95% CI)	*P*-value
SMI	Q2 vs. Q1	0.787 (0.457,1.355)	0.388
SMI	Q3 vs. Q1	0.837 (0.452,1.550)	0.572
SMI	Q4 vs. Q1	0.431 (0.227,0.818)	0.010
	*P* for trend		0.010
IMATI	Q2 vs. Q1	2.073 (1.228,3.499)	0.006
IMATI	Q3 vs. Q1	4.873 (2.865,8.289)	<0.001
IMATI	Q4 vs. Q1	5.237 (2.908,9.431)	<0.001
	*P* for trend		<0.001
MSFR	Q2 vs. Q1	0.748 (0.375,1.494)	0.411
MSFR	Q3 vs. Q1	0.564 (0.248,1.283)	0.172
MSFR	Q4 vs. Q1	0.435 (0.157,1.209)	0.110
	*P* for trend		0.089

Each parameter was split into four quartiles; Q1 = 1st quartile (lowest value, reference), Q2 = 2nd quartile, Q3 = 3rd quartile, Q4 = 4th quartile (highest value).

Inverse-probability-of-sampling weighted Cox regression with robust variance, adjusted for the same pre-specified clinical covariates as the main model [age, sex, smoking, hypertension, diabetes, BMI, TC, HDL-C, LDL-C, Lp(a), baseline lipid-lowering and anticoagulant therapy]. SMI and IMATI quartiles were modelled separately; MSFR quartiles were additionally adjusted for SMI and IMATI (continuous, per 1 SD) because MSFR is collinear with them. *P* for trend was obtained by entering the quartile rank as a continuous term. Outcomes are reported as HR (95% CI) and *P* values.

### Incremental predictive value

Adding SMI and IMATI to a model of traditional risk factors improved risk discrimination and reclassification. Harrell's C-statistic rose from 0.666 to 0.727 (*Δ*C = 0.061, 95% CI −0.009–0.132, *P* = 0.089); time-dependent AUC increased at 12, 24 and 36 months (0.599 → 0.652, 0.608 → 0.663, 0.639 → 0.700). Reclassification improved significantly (IDI = 0.044, 95% CI 0.022–0.085; continuous NRI = 0.502, 95% CI 0.320–0.669). The gain in the C-statistic did not reach statistical significance, whereas reclassification metrics and time-dependent AUC consistently favoured the body composition model (Supplementary Table S1; Supplementary Figure S2).

### Subgroup and interaction analyses

SMI and IMATI were examined across pre-specified subgroups (Supplementary Figure S3). SMI was protective in all subgroups with no significant interaction (all *P* for interaction > 0.05). IMATI was a consistent risk factor; the only significant interaction was with obesity (*P* for interaction = 0.018), with the IMATI-MACE association being stronger in non-obese (HR 1.792, 95% CI 1.380–2.326) than in obese patients (HR 1.232, 95% CI 0.968–1.568). No interaction with sex was found for either exposure, providing formal support for consistency across sexes. CAD subtype was not available and could not be tested (Supplementary Table S2).

### RCS analysis of SMI and IMATI

**Figure 4:**
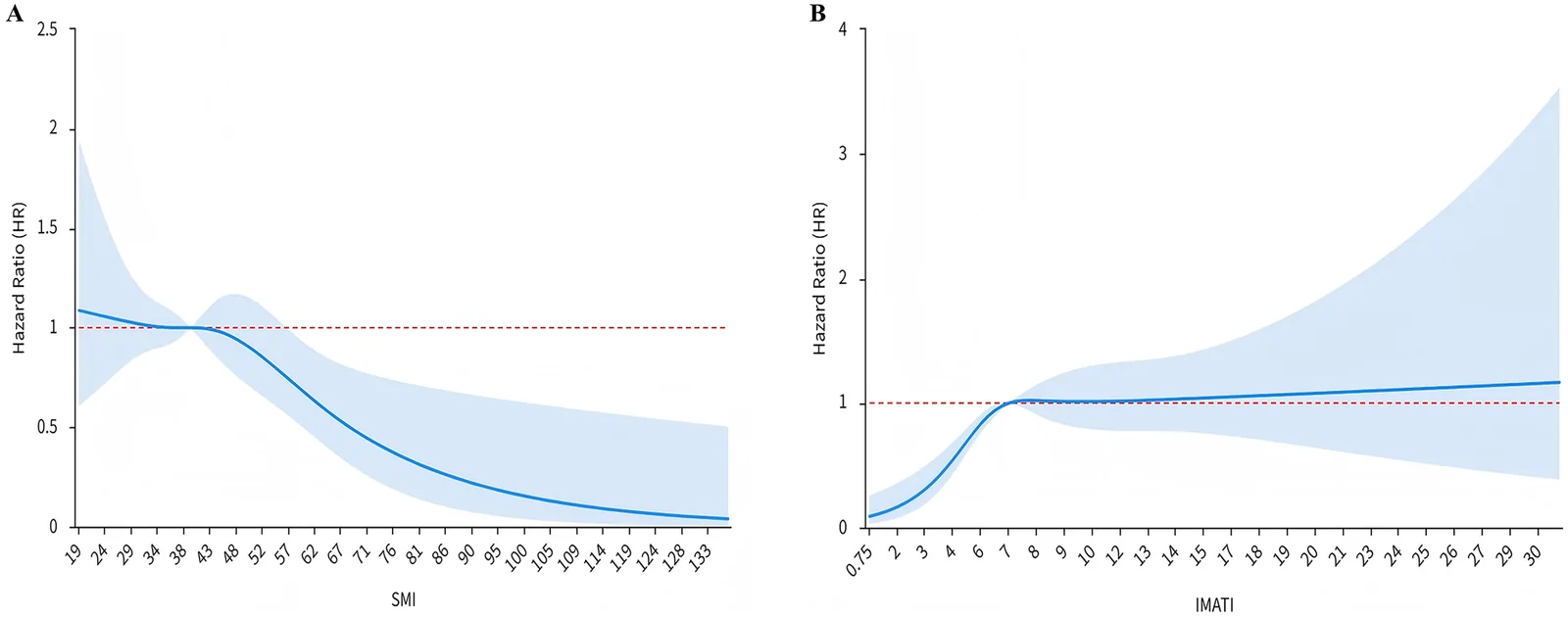
Non-linear association between body composition indices and MACE risk. **(A)** SMI; **(B)** IMATI. Legend: Blue solid line = multivariable-adjusted HR; red dashed line = reference HR = 1; blue shadow = 95%CI.

A Cox regression model with RCS, adjusted for the same pre-specified confounders, was used to further evaluate the non-linear association between SMI, IMATI and the risk of MACE (Figure 4). The results showed that SMI exhibited a non-linear downward trend in relation to MACE risk. When SMI was ≤ 39.46 cm²/m², the risk of MACE decreased as SMI increased, but the HR remained greater than 1. Once SMI exceeded this inflection point, the risk of MACE decreased significantly as SMI increased, with the HR remaining consistently below 1. Conversely, IMATI exhibited a non-linear upward trend in relation to MACE risk. When IMATI was ≤ 6.53 cm²/m², the risk of MACE increased markedly as IMATI rose, with HR values consistently below 1. Once IMATI exceeded this inflection point, the risk of MACE continued to rise with increasing IMATI, although the rate of increase slowed.

### Proportional-hazards diagnostics

The proportional-hazards assumption held for both primary exposures (scaled Schoenfeld residuals: SMI, *P* = 0.114; IMATI, *P* = 0.155; Supplementary Table S3, Supplementary Figure S4), and log-minus-log survival curves were approximately parallel (Supplementary Figure S5). The global test was significant (*P* = 0.004), driven by age (*P* = 0.047) and baseline lipid-lowering therapy (*P* = 0.006). However, in a sensitivity model stratifying the baseline hazard by these covariates, the exposure estimates were essentially unchanged (SMI HR 0.679; IMATI HR 1.788; both *P* < 0.001; Supplementary Table S4), confirming that the findings were robust to this violation.

### Assessment of consistency

The assessment results showed that external consistency between Physician 1 and Physician 2 was good, with the ICCs for SAT, SM, IMAT and VAT all exceeding 0.75. This finding indicates a high degree of agreement and reliability between the two raters regarding the assessment criteria. In the internal consistency test for Physician 1, the ICC values for SAT, SM, IMAT and VAT were also above 0.75, indicating that Physician 1 maintained a high degree of stability and consistency across multiple assessments. The specific values are shown in Supplementary Table S5.

## Discussion

This study aimed to investigate the association between body composition indices and the risk of MACE in patients with CAD. The results indicated that, compared with the non-MACE group, patients in the MACE group had higher IMATI but lower SMI. After adjusting for traditional cardiovascular risk factors, higher SMI was independently associated with a reduced risk of MACE, whereas higher IMATI was independently associated with an increased risk. RCS analysis further revealed a non-linear dose-response relationship between both indices and MACE. These findings suggest that reductions in skeletal muscle mass and increases in intramuscular fat infiltration may better reflect residual cardiovascular risk in this population than overall obesity does.

SMI is a standard metric to quantify skeletal muscle mass, and low SMI is indicative of sarcopenia (22). Accumulating cohort studies have confirmed that sarcopenia independently predicts adverse cardiovascular outcomes in patients with CAD and heart failure, including higher cardiovascular mortality and risk of repeated hospitalisation, which is consistent with our findings (23, 24). Sarcopenia reshapes the secretion pattern of muscle-derived myokines. Levels of pro-inflammatory IL-6 rise, while anti-inflammatory irisin with insulin-sensitising effects declines (25). This imbalance triggers persistent low-grade systemic inflammation and insulin resistance, which jointly accelerate atherosclerosis initiation and progression (26, 27). Skeletal muscle also serves as the primary reservoir of protein and amino acids. Muscle loss disturbs protein metabolism, amplifies oxidative stress and impairs endothelial repair capacity, further destabilising atherosclerotic plaques (28). Meanwhile, sarcopenia limits physical activity and disrupts autonomic nerve function, jointly worsening cardiovascular prognosis (29). Our restricted cubic spline analysis revealed a threshold effect of SMI at 39.46 cm²/m². The protective association of higher SMI became markedly stronger once values exceeded this inflection point, providing a concrete target for clinical nutritional and exercise intervention. In addition to the protective effect of sufficient skeletal muscle mass, intramuscular fat accumulation reflected by IMATI independently elevates cardiovascular risk via distinct metabolic and inflammatory pathways.

IMATI quantifies ectopic fat deposition within skeletal muscle, simultaneously reflecting reduced muscle quality and fat infiltration. Our results demonstrated elevated IMATI as an independent risk factor for MACE, with consistent predictive effects across both male and female subgroups, indicating IMAT infiltration as a stable cardiovascular risk marker regardless of sex. Intramuscular fat releases free fatty acids and pro-inflammatory cytokines including TNF-α and IL-6 through paracrine signalling. This forms a local lipotoxic microenvironment that damages muscle fibre function and amplifies systemic inflammation, two core drivers of atherosclerosis and plaque rupture. Even after adjusting for conventional cardiovascular risk factors, IMATI remained significantly associated with MACE risk, suggesting its pathogenic pathways are independent of general obesity and metabolic dysfunction (30, 31). Consistent with our restricted cubic spline model, IMATI exhibited a non-linear positive correlation with MACE risk. Cardiovascular risk climbed sharply when IMATI was below 6.53 cm²/m², while the growth rate of risk slowed after crossing this inflection point. This finding implies that the cardiovascular harm of intramuscular fat infiltration mainly emerges in the early accumulation stage. It is critical to distinguish IMAT from EAT, another fat depot measurable on non-contrast chest CT, as they mediate cardiovascular risk through separate anatomical and biological mechanisms (32).

Both IMAT and EAT are metabolically active fat depots that secrete pro-inflammatory adipokines, yet they promote cardiovascular injury through different routes. EAT is located directly adjacent to coronary vessels. It exerts local paracrine effects by releasing inflammatory mediators, directly stimulating coronary wall inflammation and facilitating plaque formation and destabilisation (15, 16, 33). In contrast, IMAT represents systemic ectopic fat deposition inside skeletal muscle and mainly disturbs whole-body metabolism and muscle homeostasis (34). Although both fat compartments fuel systemic inflammation, their modes of action differ distinctly: EAT acts as a local inflammatory source surrounding coronary arteries, whereas IMAT creates a persistent systemic pro-inflammatory milieu that damages blood vessels, skeletal muscle and multiple distant organs. Combined quantification of IMATI and EAT via routine non-contrast chest CT may achieve more comprehensive cardiovascular risk stratification. We did not measure EAT in this study, which constitutes a key limitation and a direction for future research. In addition to muscle and fat tissue characteristics, smoking further interacts with skeletal muscle loss to amplify MACE risk.

Smoking is a well-established modifiable cardiovascular risk factor (35). Tobacco toxicants induce endothelial dysfunction, accelerate thrombosis and exacerbate oxidative stress and inflammation, all of which accelerate atherosclerotic progression (36). In our cohort, the MACE group presented a higher smoking prevalence alongside significantly lower SMI. This coexistence suggests that smoking and sarcopenia synergistically increase cardiovascular event risk. Previous mechanistic research supports this interaction: smoking impairs mitochondrial function, accelerates muscle protein breakdown and suppresses muscle synthesis, ultimately aggravating sarcopenia (37). Clinically, combined strategies including smoking cessation and muscle-preserving training may yield synergistic benefits for reducing residual cardiovascular risk. Furthermore, SMI and IMATI retained independent predictive value after adjusting for traditional risk indicators. Opportunistic body composition evaluation based on routine non-contrast chest CT can identify high-risk CAD patients overlooked by conventional risk scoring systems and support individualised intervention plans. Despite these promising clinical implications, several limitations of the present study must be acknowledged to guide future research.

This study has several limitations. First, it was a single-centre study, which may limit generalisability. Second, 213 patients (9.6%) were lost to follow-up and 1,022 were excluded at screening by pre-defined criteria. Because individual baseline data were not retained for these patients, formal lost-versus-retained and excluded-versus-included comparisons could not be performed, and attrition and selection bias cannot be excluded (the full exclusion cascade is shown in Figure 1). The exclusion criteria were chosen to remove conditions that confound body composition measurement, supporting internal validity but restricting generalisability. Third, only baseline medication was recorded; PCSK9 inhibitors were not separately captured and were rarely used in this 2019–2021 cohort, so time-varying treatment intensity and adherence could not be modelled. Fourth, only baseline body composition was available, precluding analysis of dynamic change. Fifth, EAT was not quantified. Finally, unmeasured confounders (e.g., lifestyle, genetic background) may persist, and exploratory multiple comparisons warrant validation in independent, multicentre cohorts.

## Conclusions

In summary, after adjustment for traditional cardiovascular risk factors, higher SMI was independently associated with a lower risk of MACE in patients with CAD, whereas higher IMATI was associated with a higher risk. Chest CT-derived reductions in skeletal muscle mass and increases in intramuscular fat infiltration may therefore serve as supplementary indicators for long-term cardiovascular risk stratification, although their incremental clinical value requires validation in randomised controlled trials.

## Data Availability

The raw data supporting the conclusions of this article will be made available by the authors, without undue reservation.

## References

[1] Chong B Jayabaskaran J Jauhari SM Chia J Le Roux CW Mehta A. The global syndemic of modifiable cardiovascular risk factors projected from 2025 to 2050. J Am Coll Cardiol. (2025) 86(3):165–77. 10.1016/j.jacc.2025.04.061 40669954 PMC12718409

[2] Khan MAB Hashim MJ Mustafa H Baniyas MY Al Suwaidi SKBM AlKatheeri R. Global epidemiology of ischemic heart disease: results from the global burden of disease study. Cureus. (2020) 12(7):e9349. 10.7759/cureus.9349 32742886 PMC7384703

[3] Alkhawam H Sogomonian R El-Hunjul M Kabach M Syed U Vyas N. Risk factors for coronary artery disease and acute coronary syndrome in patients ≤40 years old. Future Cardiol. (2016) 12(5):545–52. 10.2217/fca-2016-0011 27492147

[4] Dai W Long JK Cheng Y Chen YQ Zhao SP. Elevated plasma lipoprotein(a) levels were associated with increased risk of cardiovascular events in Chinese patients with stable coronary artery disease. Sci Rep. (2018) 8:7729. 10.1038/s41598-018-25835-5 29769559 PMC5955944

[5] Fang CY Chen ZF Zhang JN Jin XQ Yang MS. Construction and evaluation of nomogram model for individualized prediction of risk of major adverse cardiovascular events during hospitalization after percutaneous coronary intervention in patients with acute ST-segment elevation myocardial infarction. Front Cardiovasc Med. (2022) 9:1050785. 10.3389/fcvm.2022.1050785 36620648 PMC9810984

[6] Silver SA Huang M Nash MM Prasad GVR. Framingham risk score and novel cardiovascular risk factors underpredict Major adverse cardiac events in kidney transplant recipients. Transplantation. (2011) 92(2):183–9. 10.1097/TP.0b013e31821f303f 21558986

[7] Cai X Liu M Xu X Zhang S Huang R Wang P. Cardiovascular effects of weight loss in old adults with overweight/obesity according to change in skeletal muscle mass. J Cachexia Sarcopenia Muscle. (2024) 15(1):342–51. 10.1002/jcsm.13409 38108096 PMC10834329

[8] Kim D Lee J Park R Oh CM Moon S. Association of low muscle mass and obesity with increased all-cause and cardiovascular disease mortality in US adults. J Cachexia Sarcopenia Muscle. (2024) 15(1):240–54. 10.1002/jcsm.13397 38111085 PMC10834318

[9] Henning RJ. Obesity and obesity-induced inflammatory disease contribute to atherosclerosis: a review of the pathophysiology and treatment of obesity. Am J Cardiovasc Dis. (2021) 11(4):504–29. 34548951 PMC8449192

[10] Lee MJ Kim J. The pathophysiology of visceral adipose tissues in cardiometabolic diseases. Biochem Pharmacol. (2024) 222(8):116116. 10.1016/j.bcp.2024.116116 38460909 PMC11407912

[11] Souza ACAH Troschel AS Marquardt JP Hadžić I Foldyna B Moura FA. Skeletal muscle adiposity, coronary microvascular dysfunction, and adverse cardiovascular outcomes. Eur Heart J. (2025) 46(12):1112–23. 10.1093/eurheartj/ehae827 39827905 PMC13376131

[12] Lin S Liu C Ding X Wang S Wu J Wang X. Body composition of metabolically unhealthy normal-weight patients with aortic stenosis: a prospective cohort study. BMC Cardiovasc Disord. (2024) 24(1):14. 10.1186/s12872-024-04400-1 39710637 PMC11665197

[13] Souza ACAH Rosenthal MH Moura FA Divakaran S Osborne MT Hainer J. Body composition, coronary microvascular dysfunction, and future risk of cardiovascular events including heart failure. JACC-Cardiovasc Imag. (2024) 17(2):179–91. 10.1016/j.jcmg.2023.07.014 PMC10922555 37768241

[14] Tolonen A Pakarinen T Sassi A Kyttä J Cancino W Rinta-Kiikka I. Methodology, clinical applications, and future directions of body composition analysis using computed tomography (CT) images: a review. Eur J Radiol. (2021) 145:109943. 10.1016/j.ejrad.2021.109943 34839215

[15] Konwerski M Gasecka A Opolski G Grabowski M Mazurek T. Role of epicardial adipose tissue in cardiovascular diseases: a review. Biology-Basel. (2022) 11(3):355. 10.3390/biology11030355 35336728 PMC8945130

[16] Napoli G Pergola V Basile P De Feo D Bertrandino F Baggiano A. Epicardial and pericoronary adipose tissue, coronary inflammation, and acute coronary syndromes. J Clin Med. (2023) 12(23):7212. 10.3390/jcm12237212 38068263 PMC10707039

[17] Saba L Loewe C Weikert T Williams MC Galea N Budde RPJ. State-of-the-art CT and MR imaging and assessment of atherosclerotic carotid artery disease: standardization of scanning protocols and measurements-a consensus document by the European society of cardiovascular radiology (ESCR). Eur Radiol. (2023) 33(2):1063–87. 10.1007/s00330-022-09024-7 36194267 PMC9889495

[18] Nam JG Goo JM. Evaluation and management of indeterminate pulmonary nodules on chest computed tomography in asymptomatic subjects. The Principles of Nodule Guidelines. Semin Respir Crit Care Med. (2022) 43(06):851–61. 10.1055/s-0042-1753474 35803268

[19] Xu K Khan MS Li TZ Gao R Terry JG Huo Y. AI body composition in lung cancer screening: added value beyond lung cancer detection. Radiology. (2023) 308(1):e222937. 10.1148/radiol.222937 37489991 PMC10374937

[20] Miller RJH Yi J Shanbhag A Marcinkiewicz A Patel KK Lemley M. Deep learning-quantified body composition from positron emission tomography/computed tomography and cardiovascular outcomes: a multicentre study. Eur Heart J. (2025) 46(24):2336–47. 10.1093/eurheartj/ehaf131 40159388 PMC12190801

[21] Zhang T Li J Li X Liu YJ. Intermuscular adipose tissue in obesity and related disorders: cellular origins, biological characteristics and regulatory mechanisms. Front Endocrinol. (2023) 14:1280853. 10.3389/fendo.2023.1280853 PMC10619759 37920255

[22] Sayer AA Cruz-Jentoft A. Sarcopenia definition, diagnosis and treatment: consensus is growing. Age Ageing. (2022) 51(10):afac220. 10.1093/ageing/afac220 36273495 PMC9588427

[23] Kang DO Park SY Park Y Jang WY Kim W Choi BG. Prognostic impact of sarcopenia on major adverse cardiovascular outcomes in coronary artery disease patients undergoing successful percutaneous coronary intervention. Eur Heart J. (2019) 40:705.30500882

[24] Fang M Liu CH Liu Y Tang G Li CL Guo L. Association between sarcopenia with incident cardio-cerebrovascular disease: a systematic review and meta-analysis. BioSci Trends. (2023) 17(4):293–301. 10.5582/bst.2023.01130 37574268

[25] Pedersen BK Febbraio MA. Muscles, exercise and obesity: skeletal muscle as a secretory organ. Nat Rev Endocrinol. (2012) 8(8):457–65. 10.1038/nrendo.2012.49 22473333

[26] Tuttle CSL Thang LAN Maier AB. Markers of inflammation and their association with muscle strength and mass: a systematic review and meta-analysis. Ageing Res Rev. (2020) 64:101185. 10.1016/j.arr.2020.101185 32992047

[27] Boström P Wu J Jedrychowski MP Korde A Ye L Lo JC. A PGC1-*α*-dependent myokine that drives brown-fat-like development of white fat and thermogenesis. Nature. (2012) 481(7382):463–U72. 10.1038/nature10777 22237023 PMC3522098

[28] Cederholm T Jensen GL Correia MITD Gonzalez MC Fukushima R Higashiguchi T. GLIM Criteria for the diagnosis of malnutrition - A consensus report from the global clinical nutrition community. J Cachexia Sarcopenia Muscle. (2019) 10(1):207–17. 10.1002/jcsm.12383 30920778 PMC6438340

[29] Zheng K Wang Z Han P Chen C Huang C Wu Y. Lower heart rate variability is associated with loss of muscle mass and sarcopenia in community-dwelling older Chinese adults. Journal of the Formosan Medical Association=Taiwan yi zhi. (2024) 123(5):571–7. 10.1016/j.jfma.2023.10.010 37996320

[30] Sachs S Zarini S Kahn DE Harrison KA Perreault L Phang T. Intermuscular adipose tissue directly modulates skeletal muscle insulin sensitivity in humans. Am J Physiol-Endocrinol Metab. (2019) 316(5):E866–79. 10.1152/ajpendo.00243.2018 30620635 PMC6580171

[31] Jang SY Choi KM. Impact of adipose tissue and lipids on skeletal muscle in sarcopenia. J Cachexia Sarcopenia Muscle. (2025) 16(4):e70000. 10.1002/jcsm.70000 40641114 PMC12246390

[32] Datta S Koka S Boini KM. Understanding the role of adipokines in cardiometabolic dysfunction: a review of current knowledge. Biomolecules. (2025) 15(5):612. 10.3390/biom15050612 40427505 PMC12109550

[33] Mancio J Azevedo D Saraiva F Azevedo AI Pires-Morais G Leite-Moreira A. Epicardial adipose tissue volume assessed by computed tomography and coronary artery disease: a systematic review and meta-analysis. Eur Heart J-Cardiovasc Imaging. (2018) 19(5):490–7. 10.1093/ehjci/jex314 29236951

[34] Fang WX Xie SY Deng W. Epicardial adipose tissue: a potential therapeutic target for cardiovascular diseases. J Cardiovasc Transl Res. (2024) 17(2):322–33. 10.1007/s12265-023-10442-1 37848803

[35] Jha P Peto R. Global Effects of Smoking, of Quitting, and of Taxing Tobacco. N Engl J Med. (2014) 370(1):60–8. 10.1056/NEJMra1308383 24382066

[36] Messner B Bernhard D. Smoking and cardiovascular disease mechanisms of endothelial dysfunction and early atherogenesis. Arterioscler Thromb Vasc Biol. (2014) 34(3):509–15. 10.1161/atvbaha.113.300156 24554606

[37] Petersen AMW Magkos F Atherton P Selby A Smith K Rennie MJ. Smoking impairs muscle protein synthesis and increases the expression of myostatin and MAFbx in muscle. Am J Physiol-Endocrinol Metab. (2007) 293(3):E843–8. 10.1152/ajpendo.00301.2007 17609255

