# Supplementary material for: Association of skeletal muscle index and intermuscular adipose tissue index measured by non-contrast chest CT with major adverse cardiovascular events in patients with coronary artery disease: a nested case-control study in a Chinese population
Source: Front Cardiovasc Med. 2026 Aug 12;13:1917241. doi: 10.3389/fcvm.2026.1917241 (PMC13507726; doi:10.3389/fcvm.2026.1917241)
Supplement: Supplementary file 1 [file Table1.docx]

**Table S1.** Incremental predictive value of SMI and IMATI over traditional risk factors

| **Metric** | **Estimate (95% CI)** |
| --- | --- |
| C-statistic, base model | 0.666 (0.578–0.754) |
| C-statistic, base + SMI + IMATI | 0.727 (0.651–0.803) |
| ΔC-statistic | 0.061 (-0.009 to 0.132), P = 0.089 |
| Time-dependent AUC, 12 mo | 0.599 → 0.652 (Δ0.053) |
| Time-dependent AUC, 24 mo | 0.608 → 0.663 (Δ0.054) |
| Time-dependent AUC, 36 mo | 0.639 → 0.700 (Δ0.061) |
| IDI | 0.044 (0.022–0.085) |
| Continuous NRI | 0.502 (0.320–0.669) |

Abbreviations: All weighted Cox models used to calculate incremental predictive metrics were adjusted for the unified pre-specified confounder panel: age, sex, smoking status, hypertension, diabetes mellitus, total cholesterol, HDL-C, LDL-C, BMI, lipoprotein(a), baseline lipid-lowering therapy and anticoagulant therapy.
