# Supplementary material for: Association of skeletal muscle index and intermuscular adipose tissue index measured by non-contrast chest CT with major adverse cardiovascular events in patients with coronary artery disease: a nested case-control study in a Chinese population
Source: Front Cardiovasc Med. 2026 Aug 12;13:1917241. doi: 10.3389/fcvm.2026.1917241 (PMC13507726; doi:10.3389/fcvm.2026.1917241)
Supplement: Supplementary file 2 [file Table2.docx]

**Table S2.** Subgroup analyses with tests for interaction (weighted Cox)

| **Exposure** | **Subgroup** | **Level** | **N** | **Events** | **HR (95% CI)** | **P** | **P-interaction** |
| --- | --- | --- | --- | --- | --- | --- | --- |
| SMI(+1SD) | Age | <65 | 253 | 126 | 0.563 (0.403–0.786) | 0.000756 | 0.136 |
| SMI(+1SD) | Age | >=65 | 297 | 149 | 0.789 (0.641–0.972) | 0.0261 | 0.136 |
| SMI(+1SD) | Sex | Female | 183 | 89 | 0.806 (0.522–1.243) | 0.329 | 0.267 |
| SMI(+1SD) | Sex | Male | 367 | 186 | 0.656 (0.526–0.817) | 0.000172 | 0.267 |
| SMI(+1SD) | Diabetes | No diabetes | 414 | 201 | 0.668 (0.538–0.828) | 0.000241 | 0.612 |
| SMI(+1SD) | Diabetes | Diabetes | 136 | 74 | 0.795 (0.532–1.187) | 0.261 | 0.612 |
| SMI(+1SD) | Obesity | BMI<28 | 372 | 190 | 0.772 (0.619–0.962) | 0.0214 | 0.225 |
| SMI(+1SD) | Obesity | BMI>=28 | 178 | 85 | 0.517 (0.329–0.813) | 0.00432 | 0.225 |
| IMATI(+1SD) | Age | <65 | 253 | 126 | 1.159 (0.924–1.453) | 0.202 | 0.264 |
| IMATI(+1SD) | Age | >=65 | 297 | 149 | 1.371 (1.127–1.670) | 0.00165 | 0.264 |
| IMATI(+1SD) | Sex | Female | 183 | 89 | 1.391 (1.019–1.900) | 0.0377 | 0.555 |
| IMATI(+1SD) | Sex | Male | 367 | 186 | 1.233 (1.035–1.469) | 0.0193 | 0.555 |
| IMATI(+1SD) | Diabetes | No diabetes | 414 | 201 | 1.233 (1.039–1.463) | 0.0164 | 0.409 |
| IMATI(+1SD) | Diabetes | Diabetes | 136 | 74 | 1.460 (1.008–2.115) | 0.045 | 0.409 |
| IMATI(+1SD) | Obesity | BMI<28 | 372 | 190 | 1.792 (1.380–2.326) | 1.2e-05 | 0.0185 |
| IMATI(+1SD) | Obesity | BMI>=28 | 178 | 85 | 1.232 (0.968–1.568) | 0.0896 | 0.0185 |

Abbreviations: All subgroup interaction weighted Cox models were adjusted for the identical pre-defined clinical covariates as the primary multivariate analysis.
