# Supplementary material for: Association of skeletal muscle index and intermuscular adipose tissue index measured by non-contrast chest CT with major adverse cardiovascular events in patients with coronary artery disease: a nested case-control study in a Chinese population
Source: Front Cardiovasc Med. 2026 Aug 12;13:1917241. doi: 10.3389/fcvm.2026.1917241 (PMC13507726; doi:10.3389/fcvm.2026.1917241)
Supplement: Supplementary file 3 [file Table3.docx]

**Table S3.** Proportional-hazards diagnostics (scaled Schoenfeld residuals).

| **Covariate** | **chi-square** | **df** | **P** |
| --- | --- | --- | --- |
| SMI_z | 2.502 | 1 | 0.114 |
| IMATI_z | 2.02 | 1 | 0.155 |
| age | 3.941 | 1 | 0.0471 |
| sex | 0.653 | 1 | 0.419 |
| smoking | 0.205 | 1 | 0.651 |
| hypertension | 0.162 | 1 | 0.687 |
| diabetes | 1.722 | 1 | 0.189 |
| TC | 3.449 | 1 | 0.0633 |
| HDL | 0.208 | 1 | 0.648 |
| LDL | 2.01 | 1 | 0.156 |
| BMI | 0 | 1 | 0.986 |
| lipid_lowering | 7.676 | 1 | 0.0056 |
| anticoagulant | 3.681 | 1 | 0.055 |
| GLOBAL | 30.193 | 13 | 0.00442 |

Abbreviations: Global P = 0.004; SMI (P = 0.114) and IMATI (P = 0.155) satisfy the assumption. Covariates tested for the proportional-hazards assumption were consistent with the full set of pre-specified adjustment factors in all Cox regression models.
