# Supplementary material for: Association of skeletal muscle index and intermuscular adipose tissue index measured by non-contrast chest CT with major adverse cardiovascular events in patients with coronary artery disease: a nested case-control study in a Chinese population
Source: Front Cardiovasc Med. 2026 Aug 12;13:1917241. doi: 10.3389/fcvm.2026.1917241 (PMC13507726; doi:10.3389/fcvm.2026.1917241)
Supplement: Supplementary file 4 [file Table4.docx]

**Table S4.** Sensitivity analysis stratifying the baseline hazard on PH-violating covariates

| **Exposure** | **HR (main model)** | **HR (stratified)** | **P (stratified)** |
| --- | --- | --- | --- |
| SMI(+1SD) | 0.667 | 0.679 | 0.000546 |
| IMATI(+1SD) | 1.916 | 1.788 | 1.4e-07 |

Abbreviations: The sensitivity stratified Cox model retained the complete pre-specified confounder panel consistent with the main analysis.
