# Supplementary material for: Association of skeletal muscle index and intermuscular adipose tissue index measured by non-contrast chest CT with major adverse cardiovascular events in patients with coronary artery disease: a nested case-control study in a Chinese population
Source: Front Cardiovasc Med. 2026 Aug 12;13:1917241. doi: 10.3389/fcvm.2026.1917241 (PMC13507726; doi:10.3389/fcvm.2026.1917241)
Supplement: Supplementary file 5 [file Table5.docx]

**Table S5** Internal consistency and external consistency

| **Variable** | **Internal Consistency** | | **External Consistency** | |
| --- | --- | --- | --- | --- |
|  | ICC | 95%CI | ICC | 95%CI |
| SATI | 0.967 | 0.919-0.987 | 0.882 | 0.727-0.952 |
| SMI | 0.974 | 0.936-0.990 | 0.833 | 0.625-0.930 |
| IMATI | 0.963 | 0.910-0.985 | 0.856 | 0.671-0.940 |
| VATI | 0.976 | 0.940-0.990 | 0.872 | 0.706-0.947 |
| MSFR | 0.969 | 0.925-0.988 | 0.845 | 0.680-0.935 |

ICC:Intraclass Correlation Coefficient

Traditional model: conventional risk factors; Body composition model: body composition indicators; Combined model: conventional risk factors + body composition indicators.
